# Configuring the Mesh Size, Side Taper and Wing Depth of Penaeid Trawls to Reduce Environmental Impacts

**DOI:** 10.1371/journal.pone.0099434

**Published:** 2014-06-09

**Authors:** Matt K. Broadhurst, David J. Sterling, Russell B. Millar

**Affiliations:** 1 NSW Department of Primary Industries, Fisheries Conservation Technology Unit, Coffs Harbour, New South Wales, Australia; 2 Sterling Trawl Gear Services, Manly, Queensland, Australia; 3 Department of Statistics, The University of Auckland, Auckland, New Zealand; Aristotle University of Thessaloniki, Greece

## Abstract

The effects of reducing mesh size while concomitantly varying the side taper and wing depth of a generic penaeid-trawl body were investigated to improve engineering performance and minimize bycatch. Five trawl bodies (with the same codends) were tested across various environmental (e.g. depth and current) and biological (e.g. species and sizes) conditions. The first trawl body comprised 41-mm mesh and represented conventional designs (termed the ‘41 long deep-wing'), while the remaining trawl bodies were made from 32-mm mesh and differed only in their side tapers, and therefore length (i.e. 1N3B or ‘long’ and ∼28^o^ to the tow direction vs 1N5B or ‘short’ and ∼35^o^) and wing depths (‘deep’–97 T vs ‘shallow’–60 T). There were incremental drag reductions (and therefore fuel savings – by up to 18 and 12% per h and ha trawled) associated with reducing twine area via either modification, and subsequently minimizing otter-board area in attempts to standardize spread. Side taper and wing depth had interactive and varied effects on species selectivity, but compared to the conventional 41 long deep-wing trawl, the 32 short shallow-wing trawl (i.e. the least twine area) reduced the total bycatch by 57% (attributed to more fish swimming forward and escaping). In most cases, all small-meshed trawls also caught more smaller school prawns *Metapenaeus macleayi* but to decrease this effect it should be possible to increase mesh size slightly, while still maintaining the above engineering benefits and species selectivity. The results support precisely optimizing mesh size as a precursor to any other anterior penaeid-trawl modifications designed to improve environmental performance.

## Introduction

Benthic otter trawling relies on hydrodynamic forces created by hydrovanes (otter boards) dragged across the seabed to achieve correct net geometry (to capture the targeted animals), and consequently is an energy intensive fishing method [1]. This is especially the case for penaeid trawls which, owing to the small sizes of the targeted species and their distributions (often buried in the soft substratum) [2], [3] require small mesh (typically 30–50 mm stretched mesh opening–SMO) and sufficient bottom contact pressure [4]. Such requirements, combined with considerable increases in oil prices over the past decade have resulted in reduced profit margins in many penaeid fisheries [5].

In addition to creating considerable drag, the spatial use (i.e. typically inshore tropical and temperate regions) of small-meshed penaeid trawls means that they generally retain disproportionate quantities of bycatch, including juveniles of commercially and recreationally important species [6]. More specifically, despite contributing towards <1.5% of the total harvest from marine capture fisheries, penaeid trawling is responsible for approximately one quarter of global discards [5], [7]; much of which is associated with considerable mortality and the implicit assumption of negative impacts on some stocks [8].

The above concerns over high energy intensities and poor size and species selectivities of penaeid trawls have mostly been separately assessed; typically through on-going industry-based efforts at improving operational efficiencies [9], and more collaborative work with scientists to develop physical modifications to codends (posterior sections of trawls) that improve selectivity (i.e. bycatch reduction devices –BRDs) [10]. However, both of these issues might be concomitantly addressed by modifying the anterior trawl section (or rigging), including the: number of trawls (i.e. single- or multi-trawl systems) [6], [11]; spreading mechanisms [9]–[13]; body and frame-line tapers [14], [15]; and especially the material, twine diameter and size of mesh [16]–[18]. The mesh characteristics are particularly important, since not only do the lateral openings ultimately influence what escapes or is retained (for small animals), the twine typically comprises >70% of the total system area for most penaeid trawling systems, and therefore strongly affects drag.

In many cases, changing any of the above parameters within the anterior trawl section will influence catching and engineering performances, although there are clearly dominant factors and often complex and interactive relationships [11]–[13], [15], [17]. For example, in a recent study in an Australian penaeid-trawl fishery, we demonstrated the utility of shorter trawls (via increasing body taper from 1N2B to 1N5B, or the netting angle to the direction of towing from ∼25 to 35^o^) for significantly reducing the bycatch of one teleost (southern herring *Herklotsichthys castelnaui*) by up to 66% and also drag [15], although there was also some loss of penaeids (school prawns *Metapenaeus macleayi*). The lower school prawn catches were hypothesized to be caused by an increase in their collision probability (i.e. more acute angles of netting) against too large a mesh (legal mesh size is ≥40 mm SMO in this fishery) for the targeted sizes (mean carapace lengths–CL of >∼15 mm) [19]. By comparison, most southern herring were larger than the mesh and probably escaped more easily either through the posteriorly located BRD, or from the mouths of the shorter trawls [15].

These results highlight the need to ensure the most appropriate mesh size and/or rigging arrangements to maintain consistent lateral openings in trawl bodies as a precursor to examining other changes designed to improve selectivity and reduce drag. However, it is also important to consider that irrespective of the mesh size, various design factors also affect lateral openings [16]. One potentially important variable is the area of netting in the trawl wings (typically controlled by depth or the number of meshes in the transverse direction), which varies considerably among designs. Because the headline height of many penaeid trawls is largely determined by the height of the otter boards, unlike fish trawls, varying wing depth will not necessarily affect the vertical trawl opening, but concomitant differences in associated twine area should impact on drag and potentially selectivity [16].

Given the above, this study sought to contribute to recent efforts aimed at holistically reducing the environmental impacts of penaeid trawling through modifications to the anterior section, by assessing the utility of more closely matching mesh size to the targeted species while concomitantly examining the importance of body taper and twine area in the wings as means for reducing drag and improving species and size selectivity. The work was done within one Australian penaeid-trawl fishery, but the results are applicable to international fisheries.

## Materials and Methods

### Ethics statement

The research was done in Lake Wooloweyah (29°26′ S 153°22′ E) and the Clarence River (29°27′ S 153°12′ E) New South Wales (NSW) Australia and in accord with the Department of Primaries Industries scientific collection permit (No. P01/0059(A)-2.0). No specific permissions were required for the locations. The field studies did not involve endangered or protected species. Animal ethics approval for the research was granted by the NSW DPI Animal Care and Ethics Committee (Ref. 08/06).

### Location and vessel

**Figure 1:**
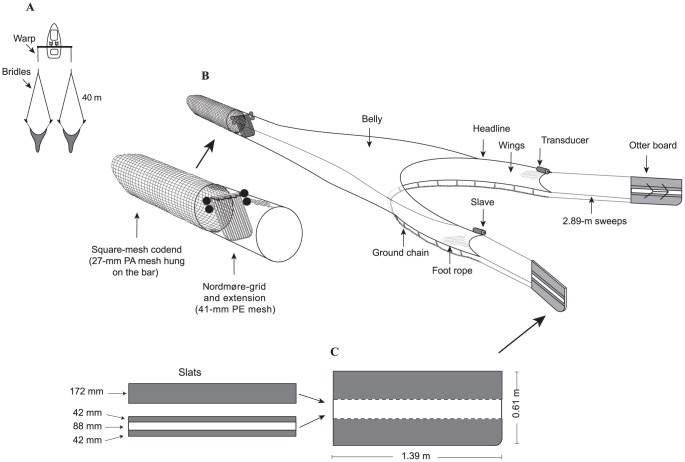
Diagrammatic representation of the (A) double-rig configuration, (B) 32 long shallow-wing trawl, and enlarged Nordmøre-grid and square-mesh codend and (C) otter boards with two removable slots. PA, polyamide; and PE, polyethylene.

The work involved two experiments completed between October 2012 and April 2013 in Lake Wooloweyah (experiment 1) and the Clarence River (experiment 2), using a double-rigged trawler (10 m and 89 kw) fishing in ∼1–18 m across sandy and mud substrata (Figure 1a). The vessel had two winch drums; each holding 8-mm diameter–Ø stainless warps and 40-m bridles (6-mm Ø stainless wire) (Figure 1a). The trawler was also equipped with: a fuel monitor (Floscan series 9000); global positioning system (GPS; Lowrance); hull-mounted sum log (EchoPilot, Bronze Log+), warp-attachable load cells and associated data logger (Amalgamated Instrument Company; model nos PA6139 and TP4); and a portable acoustic, trawl-monitoring system with wing-end distance sensors (Notus Trawlmaster System; Model no. TM800ET) [13].

### Trawl configurations and otter boards

**Figure 2:**
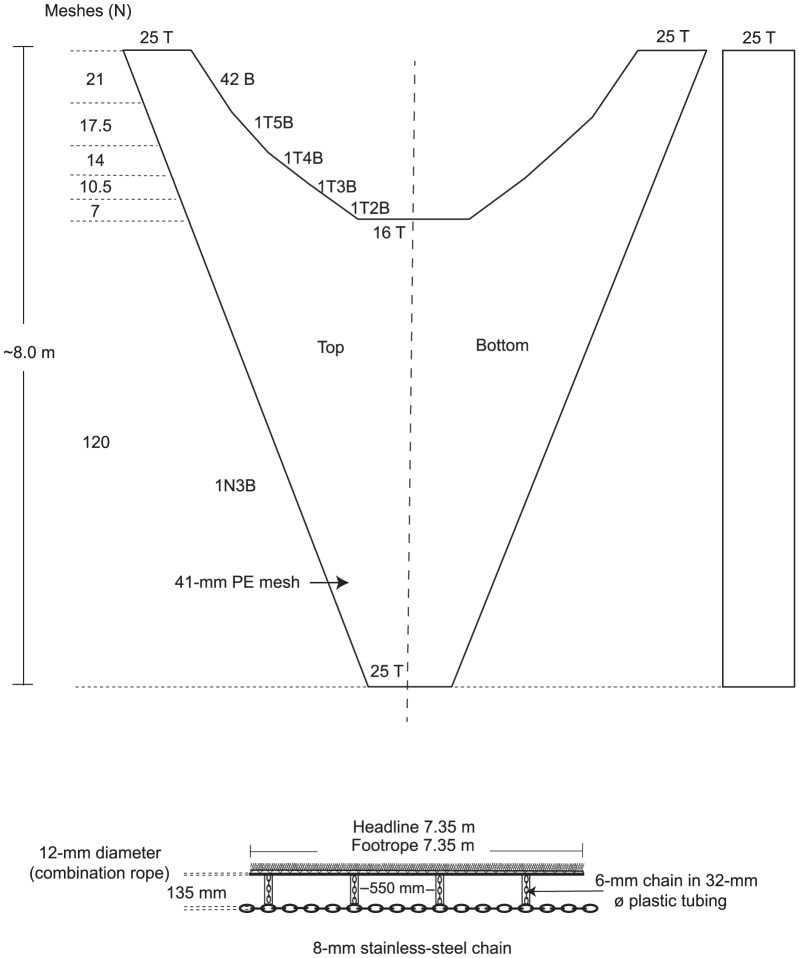
Plan of the conventional 41 long deep-wing trawl. N, normal; T, transversals; B, Bars; and Ø, diameter.

Five trawl bodies (encompassing wings and belly) were assessed; all with the same headline and footrope lengths (7.35 m), ground gear configurations, sweeps (2.89 m) and identical color and twine material, and length-for-length clusters of headline and foot-rope tapers (Figures 1 and 2, Table 1). One of the trawl bodies was a conventional design used in the Clarence River and made from nominal 41-mm (stretched mesh opening–SMO) mesh (1.20-mm Ø twine) with a side taper of 1N3B and termed the ‘41 long deep-wing’ (Figures 1 and 2, Table 1). The remaining four trawl bodies were constructed from nominal 32-mm (SMO) mesh and narrower 0.88-mm Ø twine (to maintain a constant twine-Ø-mesh-size ratio among designs) and differed only in their wing depths (deep−97 vs shallow−60 T) and side tapers (long−1N3B vs short−1N5B) (Figure 2, Table 1). The four smaller-mesh trawls were termed the (1) ‘32 long deep-wing’ (same dimensions as the 41-long deep-wing trawl including twine area, but smaller mesh); (2) ‘32 long shallow-wing’; (3) ‘32 short deep-wing’; and (4) ‘32 short shallow-wing’ (Figures 1 and 2, Table 1). All trawl bodies had the same knot directions providing up force on the top and bottom panels and out force on the side panels and were rigged with Nordmøre-grid BRDs in nominal 41-mm extension sections and square-mesh codends made from nominal 27-mm polyamide mesh hung on the bar (Figure 1b).

**Table 1:** Technical specifications of the trawls and rigging.

		41 long deep-wing	32 long deep-wing	32 long shallow-wing	32 short deep-wing	32 short shallow-wing
Trawl body	Stretched mesh opening (mm)	41.33	31.60	31.60	31.60	31.60
	Twine diameter (mm)	1.10	0.88	0.88	0.88	0.88
	Wing end (T)	75	97	60	97	60
	Wing end (stretched m)	∼3.20	∼3.20	∼1.92	∼3.20	∼1.92
	Headline and footrope tapers (half trawl)	42 AB; 7 × (1T5B; 1T4B; 1T3B and 1T2B); and 16 T	54 AB; 9 × (1T5B; 1T4B; 1T3B; and 1T2B); and 21 T	54 AB; 9 × (1T5B; 1T4B; 1T3B; and 1T2B); and 21 T	54 AB; 9 × (1T5B; 1T4B; 1T3B and 1T2B); and 21 T	54 AB; 9 × (1T5B; 1T4B; 1T3B; and 1T2B); and 21 T
	Body/wing side tapers	1N3B	1N3B	1N3B	1N5B	1N5B
	Wing length (N)	70	90	90	90	90
	Belly length (N)	120	155	124	116	90
	Belly length (m)	∼5.1	∼5.1	∼4.1	∼3.8	∼2.9
	Posterior body circumference (T)	150	194	194	194	194
	Weight (kg)	27.60	29.32	28.23	28.41	27.50
	Total twine area (m^2^)	4.80	4.95	3.64	3.86	2.75
Otter boards	Individual area (m^2^) used in the lake	0.85	0.85	0.85	0.85	0.85
	Individual weight (kg) used in the lake	51.90	51.90	51.90	51.90	51.90
	Individual area (m^2^) used in the river	0.85	0.85	0.73	0.73	0.61
	Individual weight (kg) used in the river	51.90	51.90	50.60	50.60	49.30
Total system	Area (m^2^) used in the lake	7.07	7.22	5.91	6.12	5.02
	Weight (kg) used in the lake	133.68	135.40	134.31	134.49	133.58
	Area (m^2^) used in the river	7.07	7.22	5.67	5.88	4.54
	Weight (kg) used in the river	133.68	135.40	131.71	131.89	128.38

Each trawl body was attached to an extension section with a Nordmøre-grid installed and codend (total weight of 5.80 kg) and had a catch surface area of 0.23 m^2^. Twine areas were calculated for those surfaces that were at an angle to the direction of towing (i.e. do not include the parallel panels of netting in the codend or extension). Total system area is the sum of the twine, ground-chain (0.073 m^2^), two otter boards, six sweep (0.09 m^2^), two frame-line (0.18 m^2^) and catch areas for each trawl. The total weight of all sweeps was 2.28 kg.

T, transversals; N, normal; B, bars.

The four otter boards were flat rectangular (1.39×0.61 m) and, via two removable slotted timber planks, adjustable to three surface areas (0.85, 0.73 and 0.61 m^2^; Figure 1c, Table 1). During experiment 1, the water was shallow (<2 m) and the trawls were deployed with only ∼10-m of bridle (Figure 1a). Therefore to achieve sufficient spread at the high bridle angle, the largest area (0.85 m^2^) otter boards were used with all trawls (Figure 1c, Table 1). In contrast, during experiment 2 in the deeper (mostly 10–18 m) Clarence River, 40-m bridles were deployed for all hauls and otter-board areas were configured in an attempt to achieve similar (and optimal) wing-end spreads among the different trawls (Figure 1a and c, Table 1).

### Experimental design and data collected

Prior to testing, the five trawls were weighed and 15 randomly-selected meshes were measured using a local, purpose-built mesh gauge in the bodies, extensions and codends for SMO (Table 1). On each fishing day, the trawls being tested were alternately attached to the sweeps and relevant otter boards on each side of the vessel, and the Notus distance sensors and slaves secured at the ends of the inner and outer wings (Figure 1b). After each trawl was deployed, the load cells were attached to the towing wires.

There were 10 possible paired combinations of the five trawls, but it was only practical to complete five deployments (40-min) on each day. Therefore, in each experiment, we assessed all combinations over two-day blocks, providing two daily replicates of each trawl on each day. Over seven and twelve days of fishing in Lake Wooloweyah and the Clarence River, this provided 14 and 24 replicate deployments (attempting conventional target SOGs of ∼1.20 ms^−1^) of each trawl, with an even distribution of treatments between sides of the vessel.

The technical data collected during each deployment included the: (1) drag (kgf) of each configuration; (2) the total distance trawled (otter boards on and off the bottom – obtained from the plotter and trawl-monitoring system); (3) speed the ground (SOG) and through the water (STW; both in ms^−1^), (4) depth of fishing, (5) distance of the trawls behind the vessel, and (6) the wing-end spreads (all in m). All data were recorded at 60-s intervals. During experiment 1, the shallow water and interference from the propeller wash precluded simultaneous data acquisition from the paired trawls by the hydrophone, and so it was positioned in front of each trawl for half the deployment (i.e., ∼20-min of wing-end spread data for each trawl). Both trawls were simultaneously monitored throughout the deployments in experiment 2.

Biological data were also collected at the end of each deployment and included the: total weights of school prawns and bycatch; numbers of each bycatch species; and total lengths (TL to the nearest 05 cm) of the most abundant teleosts. Random samples of ∼500 g of school prawns were placed into plastic bags and transferred to the laboratory, where they were measured (CL in mm), weighed and counted. The latter data were used to estimate the numbers and weights of ‘optimal’ commercial (≥15 mm CL) and sub-commercial (<15 mm CL) school prawns during each deployment.

### Statistical analyses

The hypothesis of no differences in the mesh sizes of the various trawls, extensions and codends was tested in separate linear models (LM), and any significant differences subsequently explored using the Benjamini-Hochberg-Yekutieli procedure to control the false discovery rate (FDR) [20]. Within each experiment, the remaining technical and biological data were analyzed in linear mixed models (LMMs), with some standardized prior to analyses. To remove any confounding effects of slightly different wing-end spreads (see Results), the numbers and weights of catches were standardized to per ha trawled using the swept area of the foot rope (calculated by average wing-end spread × the distance trawled) and then log-transformed so that differences between gears would act multiplicatively rather than additively. Predicted mean numbers and weights (per ha) were obtained by back-transforming. All other data, including the mean CL per deployment, drag, wing-end spread, SOG, STW and distance trawled were analyzed in their raw form.

All models included ‘trawl’ as fixed, while ‘trawl sides’ and ‘days’ and the interaction between ‘deployments’ and days were included as random terms. For the LMM assessing drag and spread, additional random terms involved load cells and the paired Notus sensors, respectively while additional covariates included SOG, ‘current’ (calculated as the speed of the water in the direction of travel and defined as SOG–STW), distance aft of the trawl configuration from the vessel and fishing depth. All models were fitted using the lmer function from the lme4 package of the R statistical language and the significance of trawl design was determined using a likelihood ratio test (LRT). Significant differences were investigated using FDR pair-wise tests.

Predicted means from the LMMs for drag were used to calculate relative fuel consumption associated with towing the five trawls. Specifically, assuming that for any given towing speed, the concomitant fuel usage was proportional to the drag, it is possible to determine relative fuel consumption rate using the predicted mean drags. Fuel consumption was standardized to per ha trawled for each trawl design by comparing the fuel consumption rate with predicted mean wing-end spread (the rate at which area was being swept for a given trawl speed) from the relevant LMMs.

## Results

There was a significant difference in SMO between the 41-conventional trawl (mean ±SE of 41.43±0.11 mm) and the four smaller-meshed designs (LM, *p*<0.001), but no significant differences among the latter (FDR, *p*>0.05; overall mean ±SE of 31.61±0.08 mm). There were no significant differences in SMO among the extensions (41.50±0.24 mm) and codends (27.37±0.10 mm) (LM, *p*>0.05).

Pooled across experiments, the five trawls caught 1511 and 119 kg of school prawns and bycatch (Table 2). The total bycatch included 31 species but in experiment 1, tailor *Pomatomus saltatrix* (ranging in size from 4.0–20.0 cm TL), Ramsey's perchlet *Ambassis marianus* (5.0–11.0 cm TL), and southern herring *Herklotsichthys castelnaui* (6.0–17.0 cm TL) comprised 85% of catches, while in experiment 2, forktail catfish *Arius graeffei* (7.0–19.0 cm TL) represented 83% of catches. These three species, along with school prawns, form the basis of the biological analyses (Table 2).

**Table 2:** Scientific and common names and numbers of organisms caught during experiments 1 and 2.

Group	Family	Scientific name	Common name	Experiment 1	Experiment 2
*Crustaceans*	Penaeidae	*Metapenaeus macleayi*	School prawn	120 304	424 406
	Palaemonidae	*Macrobrachium* sp.	Freshwater prawn	–	19
*Molluscs*	Loliginidae	*Uroteuthis* sp.	Squid	44	–
*Teleosts*	Ambassidae	*Ambassis jacksoniensis*	Port Jackson glassfish	148	14
		*Ambassis marianus*	Ramsey's perchlet	1 639	48
	Anguillidae	*Anguilla australis*	Southern shortfin eel	–	7
	Apogonidae	*Siphamia roseigaster*	Pink-breasted siphonfish	298	–
	Ariidae	*Arius graeffei*	Forktail catfish	1	3 008
	Clupeidae	*Herklotsichthys castelnaui*	Southern herring	1 298	217
		*Hyperlophus vittatus*	Whitebait	16	9
	Engraulidae	*Engraulis australis*	Australian anchovy	13	1
	Enoplosidae	*Enoplosus armatus*	Old wife	–	1
	Gerreidae	*Gerres subfasciatus*	Silver biddy	330	2
	Hemiramphidae	*Hyporhamphus regularis*	River garfish	8	–
	Megalopidae	*Megalops cyprinoides*	Oxeye herring	–	2
	Mugilidae	*Liza argentea*	Goldspot mullet	31	–
		*Mugil cephalus*	Sea mullet	9	–
	Muraenesocidae	*Muraenesox bagio*	Common pike eel	–	2
	Plotosidae	*Euristhmus lepturus*	Longtail catfish	5	–
	Paralichthyidae	*Pseudorhombus arsius*	Largetooth flounder	28	–
	Percichthyidae	*Macquaria novemaculeata*	Australian bass	–	1
	Platycephalidae	*Platycephalus fuscus*	Dusky flathead	31	4
	Pomatomidae	*Pomatomus saltatrix*	Tailor	4 864	3
	Sciaenidae	*Argyrosomus japonicus*	Mulloway	2	–
	Sillaginidae	*Sillago ciliata*	Sand whiting	64	2
	Soleidae	*Synclidopus macleayanus*	Narrow-banded sole	–	119
	Sparidae	*Acanthopagrus australis*	Yellowfin bream	304	91
		*Rhabdosargus sarba*	Tarwhine	35	–
	Tetraodontidae	*Tetractenos glaber*	Toadfish	4	–
	Tetrarogidae	*Centropogon australis*	Fortescue	–	1
		*Notesthes robusta*	Bullrout	–	79
	Urolophidae	*Trygonoptera testacea*	Stingray	2	–

–, not present in catches.

### Experiment 1: Lake Wooloweyah

The five trawl designs were towed at SOGs of between 1.17 and 1.43 ms^−1^, covering distances of between 3.12 and 3.87 km. There were significant differences in wing-end spread and drag among trawls (LMMs, *p*<0.001; Tables 3 and 4). The only appropriate fixed effect in the LMM for wing-end spread was trawl, with the predicted mean (±SE) wing-end spread of the 32 short shallow-wing trawl (4.47±0.05 m) significantly greater than both the 41 (4.29±0.05 m) and 32 long deep-wing trawls (4.27±0.06 m) (FDR, *p*<0.05; Table 4). No significant differences were detected among the wing-end spreads of the above trawls and either the 32 long shallow-wing (4.40±0.05 m) or short deep-wing trawls (4.31±0.05 m) (FDR, *p*>0.05; Table 4).

**Table 3:** Summaries of likelihood ratio test (LRT) statistics from linear mixed models assessing the importance of the fixed effect of trawl (conventional 41 long deep-wing, and 32 long deep- and shallow-wing and 32 short deep- and shallow-wing trawls) in explaining variability among technical and biological responses.

Variables		Experiment 1	Experiment 2
Technical	Wing-end spread	11.52*	95.24***
	Drag	20.39**	177.29***
Biological	Wt of commercial school prawns ha^−1^	21.25***	14.93**
	No. of commercial school prawns ha^−1^	21.99***	17.26**
	Wt of sub-commercial school prawns ha^−1^	52.28***	20.69***
	No. of sub-commercial school prawns ha^−1^	53.15***	20.92***
	Mean CL of school prawns	23.96***	19.43***
	Wt of total bycatch ha^−1^	33.90***	22.47***
	No. of total bycatch ha^−1^	42.73***	12.75*
	No. of tailor ha^−1^	48.24***	–
	No. of Ramsey's perchlet ha^−1^	8.58	–
	No. of southern herring ha^−1^	53.60***	–
	No. of fork tail catfish ha^−1^	–	8.29

Excluding the mean CL of school prawns (*Metapenaeus macleayi*), all other numbers and weights were standardized to per ha trawled, calculated using the total average wing-end spread (per deployment) and then log-transformed.

–, not present in catches,*p<0.05, **p<0.01, ***p<0.001.

**Table 4:** Summary of predicted mean ±SE wing-end spreads (m) and drags (kgf) and subsequent estimated fuel rates and intensities for the five trawl designs tested in experiments 1 (Lake Wooloweyah) and 2 (Clarence River).

		41 long deep-wing	32 long deep-wing	32 long shallow-wing	32 short deep-wing	32 short shallow-wing
Experiment 1	Wing-end spread (m)	4.29 (0.05)^A^	4.27 (0.06)^A^	4.40 (0.05)^A,B^	4.31 (0.05)^A,B^	4.47 (0.05)^B^
	Drag (kgf)	256.14 (1.82)^B^	256.89 (1.82)^B^	248.48 (1.80)^A^	258.57 (1.80)^B^	251.83 (1.81)^A^
	Fuel rate (L h^−1^)	5.29	5.30	5.13	5.34	5.20
	Fuel intensity (L ha^−1^)	2.64	2.66	2.49	2.65	2.49
Experiment 2	Wing-end spread (m)	5.23 (0.03)^C^	5.22 (0.03)^C^	5.08 (0.03)^B^	4.91 (0.03)^A^	4.89 (0.03)^A^
	Drag (kgf)	269.38 (1.57)^D^	270.49 (1.58)^D^	242.79 (1.61)^B^	246.59 (1.61)^C^	220.59 (1.65)^A^
	Fuel rate (L h^−1^)	6.13	6.16	5.53	5.61	5.02
	Fuel intensity (L ha^−1^)	2.67	2.69	2.48	2.60	2.34

Mean predicted drags were derived with a centred value of speed across the ground and with zero current.

Dissimilar superscript letters for wing-end spread and drag within experiments indicate significant differences detected in false-discovery-rate pairwise comparisons (*p*<0.05).

The LMM for drag included the fixed effects of both trawl and SOG, and so predicted means are presented at the centred value of SOG (i.e. drag at average SOGs; Table 4). There was no significant difference between the drags of the two shallow-wing trawls (FDR, *p*>0.05), but both had significantly lower drags (by 1.7–3.9%) than the three deep-wing trawls (FDR, *p*<0.05;Table 4). These differences were reflected in fuel rates and intensities that varied by up to 0.21 L h^−1^ and 0.17 L ha^−1^ (Table 4). Irrespective of the trawl, there was an overall positive relationship between SOG and drag (LMM, *p*<0.001).

**Figure 3:**
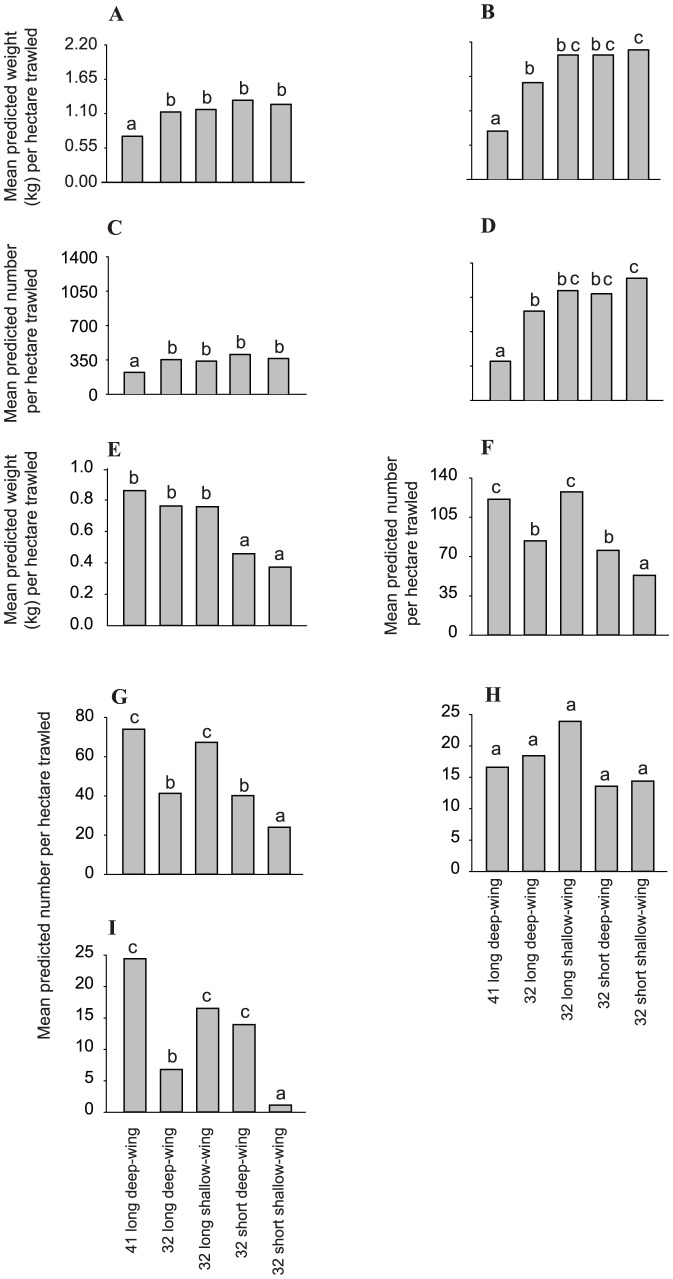
Differences in predicted mean catches per ha trawled between the conventional 41 long deep-, and 32 long deep- and shallow-wing, and 32 short deep- and shallow-wing trawls used in experiment 1 (Lake Wooloweyah) for the weights of (A) commercial and (B) sub-commercial school prawns (*Metapenaeus macleayi*) numbers of (C) commercial and (D) sub-commercial school prawns, (E) weights and (F) numbers of total bycatch, and numbers of (G) tailor *Pomatomus saltatrix*, (H) Ramsey's perchlet *Ambassis marianus*, and (I) southern herring *Herklotsichthys castelnaui*. Dissimilar letters above the histograms indicate significant differences detected in false-discovery-rate pairwise comparisons (*p*<0.05).

**Figure 4:**
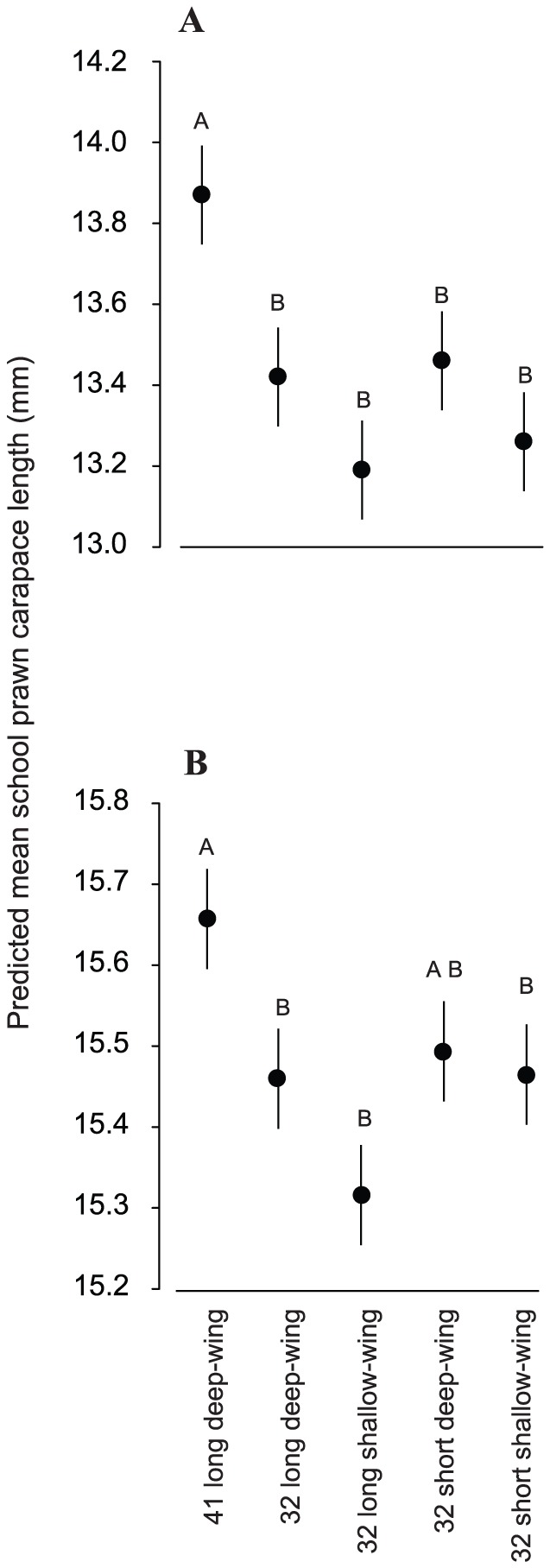
Predicted mean (±SE) carapace lengths in mm retained by the five trawls in (A) experiment 1 (Lake Wooloweyah) and (B) experiment 2 (Clarence River).

For the biological variables, compared to the conventional 41 long deep-wing trawl, all small-meshed trawls caught significantly more commercial (by up to double) and sub-commercial (by up to almost three times) school prawns per ha trawled (Figure 3a−d); which when considered across total catches manifested as significantly smaller mean sizes of individuals (LMM and FDR, *p*<0.05; Figures 3a–e, g, 4a, Table 3). Conversely, irrespective of their wing depth, the two 32 short trawls caught significantly less total bycatch by weight per ha than the other three trawls (by between 40 and 57%; LMM and FDR, *p*<0.05; Figure 3c, Table 3). Further, compared to all trawls, the 32 short shallow-wing trawl caught significantly fewer (per ha) total bycatch (by 29–57%), southern herring (by 83–95%), and tailor (by 40–67%) by number (LMM and FDR, *p*<0.05; Figure 3d–f, Table 3). Differences in the above variables among the other trawls were less consistent (Fig. 3d–f). There were no significant differences between trawls for the number of Ramsey's perchlet per ha trawled (LMM, *p*>0.05; Fig. 3f, Table 3).

### Experiment 2: Clarence River

During 12 days of fishing, the five trawls were towed at SOGs of 1.00–2.11 ms^–1^, over distances of 2.28–5.07 km. Wing-end spreads and drags were significantly different among trawls (LMMs, *p*<0.001; Tables 3 and 4). Broadly, the predicted mean (±SE) wing-end spreads were separated into three groups, with the 32 short shallow- and deep-wing trawls fishing significantly narrower (4.91±0.03 m and 4.89±0.03 m, respectively) than the 32 long shallow-wing trawl (5.08±0.03 m); which in turn was spread significantly less than both the 41- and 32 long deep-wing trawls (5.23±0.03 and 5.22±0.03 m, respectively) (FDR, *p*<0.05; Table 4).

The LMM for drag included the fixed effects of trawl, SOG and current (*p*<0.001; Table 3). The average current relative to the heading of the trawler was only 0.03 ms^−1^, and so the predicted means were presented at the centred value of SOG (Table 4). The 32 short shallow-wing trawl had the significantly lowest drag (by between 9.4 and 18.4%) followed by the 32 long shallow-wing, short deep-wing and both the long deep-wing trawls (FDR, *p<*0.05; Table 4). The above differences corresponded to variations in fuel rates and intensities among trawls by up to 1.14 L h^−1^ and 0.35 L ha^−1^ (Table 4). Irrespective of trawl, there were overall positive relationships between drag and both SOG and current (LMM, *p*<0.001).

**Figure 5:**
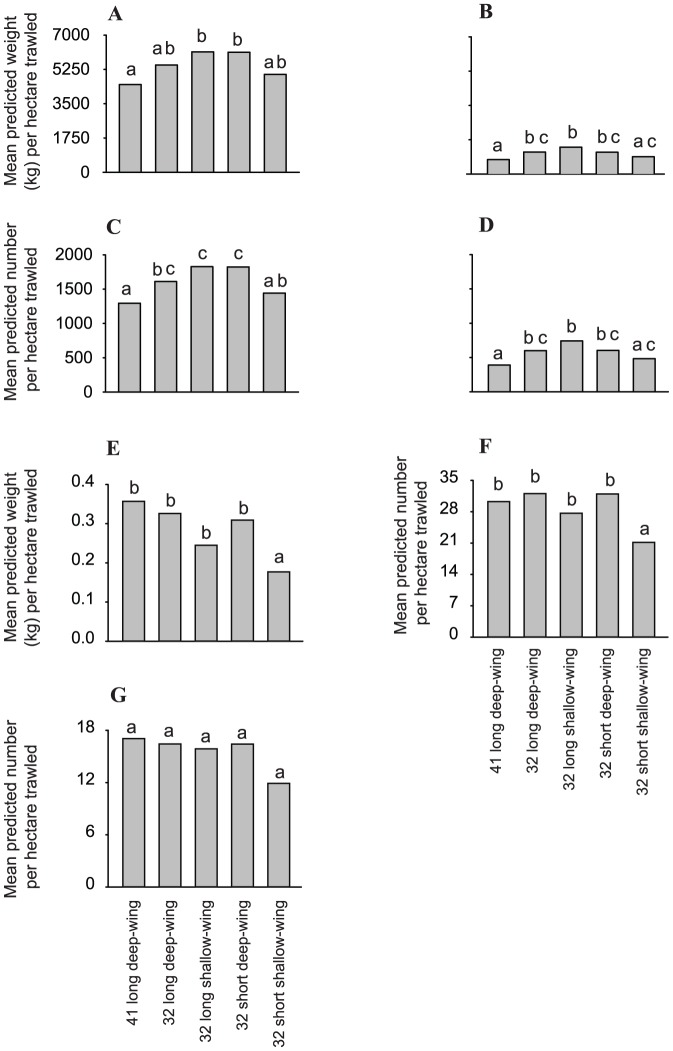
Differences in predicted mean catches per ha trawled between the conventional 41 long deep-, and 32 long deep- and shallow-wing, and 32 short deep- and shallow-wing trawls used in experiment 2 (Clarence River) for the weights of (A) commercial and (B) sub-commercial school prawns *Metapenaeus macleayi*, numbers of (C) commercial and (D) sub-commercial school prawns, (E) weights and (F) numbers of total bycatch, and (G) number of forktail catfish *Arius graeffei*. Dissimilar letters above the histograms indicate significant differences detected in false-discovery-rate pairwise comparisons (*p*<0.05).

For catches, the four small-meshed trawls generally caught similar quantities of commercial and sub-commercial school prawns per ha trawled, and mostly significantly more than the conventional 41 long deep-wing trawl (by up to 1.4 and 1.9 times more, respectively) (LMM and FDR, *p*<0.05; Figure 5a–d, Table 3). The obvious exception was the 32 short shallow-wing trawl, which caught the same quantities of school prawns per ha across both categories as the conventional 41 long deep-wing trawl (LMM and FDR, *p*>0.05; Figure 5a−d). Combined across all catches, the mean size of school prawns retained by the 41 long deep-wing trawl was significantly larger than all small-mesh trawls (LMM and FDR, *p*<0.05); except the 32 short deep-wing trawl (Figure 4b). Compared to all other trawls, the 32 short shallow-wing trawl caught significantly less bycatch by both weight (26.25–50.42%) and number (23.60–34.01%) (LMM and FDR, *p*<0.05; Figure 5c and d, Table 3). There were no significant effects of trawl on forktail catfish numbers (LMM, *p*>0.05; Figure 5e, Table 3).

## Discussion

The results from this study further highlight the potential for improving the engineering and biological performances of penaeid trawls simply by changing their anterior mesh (1) lateral openings (via different SMOs and wing twine areas) and (2) orientation angles (via different body tapers) [11], [13], [14], [17]. By replicating the work across two experiments characterized by divergent environmental (e.g. water depth and current) and biological (e.g. different sizes of the targeted species and species assemblages) conditions, we have also provided some indication of the relative importance of such extrinsic variables on the key gear changes. The complex interaction of these variables can be discussed with respect to probable gear geometry and associated species-specific responses, and ultimately used to suggest further refinements.

Changing the mesh size, but concomitantly scaling the twine diameter, ensured that there was no appreciable difference in the twine areas of the 41 and 32 long deep-wing trawls, and both were spread using the same otter boards. Consequently, in each experiment, these trawls had almost identical wing-end spreads, drag, and therefore required similar fuel per h and ha trawled. As might be expected, the smaller-meshed trawl retained significantly smaller school prawns (although the larger-meshed trawl also lost considerable quantities of commercial-sized individuals); a trend that was maintained across clearly divergent size distributions (i.e. much smaller school prawns in Lake Wooloweyah than in the Clarence River). But, despite the much smaller mesh, the 32 long deep-wing trawl caught significantly fewer tailor (by 56%) and southern herring (by 72%), contributing to less total bycatch (by 31%) than the 41 long deep-wing trawl in the lake. The same pattern was not observed in in the river (for the only species caught in abundance—forktail catfish).

Because most of the tailor (4.0–20.0 cm TL) and southern herring (6.0–17.0 cm TL) had girths larger than the perimeter of the 32-mm mesh [15], many of the observed differences in catches may reflect either trawl avoidance completely, or potentially greater escape back through the mouth after entering. Both of these mechanisms might be attributed to the relative visibility of the trawls and/or species-specific responses to associated stimuli. More specifically, the lake was shallow (<2 m) and the relatively larger numbers of fish may have been more easily able to detect the smaller-meshed trawl and perhaps either avoid capture. By contrast, forktail catfish may have been less able to detect the approaching trawls in the much deeper river (3–18 m), although previous studies showed that this species had limited response to other anterior trawl body changes [11], [13].

While not as extreme as a reduction in mesh size, varying wing depth probably also affected lateral openings in the smaller-meshed trawl bodies, although the influence on catches appeared to at least partially depend on the side taper. It is clear that steepening the side taper (in this case from 1N3B to 1N5B) causes at least two geometric changes, including (1) shortening the length of the trawl body and (2) increasing the angle of netting to the direction of tow. In terms of teleost bycatch, both effects are probably of consequence. Specifically, in an earlier study [15] we attributed bycatch reductions in other shorter trawl bodies tested in this fishery to some fish detecting the trawl and then because of the slightly reduced distance, more easily escaping (especially during haul back) [21]; either back out through the mouth (as proposed for other teleosts) [14], or through the opening at the top of the Nordmøre-grid. Similar escape mechanisms may have occurred here.

In Lake Wooloweyah, both shorter, small-meshed trawls caught significantly less bycatch (by weight) than the longer designs, supporting the trend above, however, within side taper there were divergent effects of wing depth. In particular, the 32 long deep-wing trawl caught fewer southern herring, tailor and therefore total bycatch than the 32 long shallow-wing trawl, while the opposite relationship occurred for the 32 short trawls for these species. Such results are difficult to explain, although given there were only small variations in predicted wing end spread (e.g. <5% differences) and assuming comparable vertical fishing heights and ground gear contact, two possible effects were the relative visibilities of the trawls in the shallow lake and species-specific escape mechanisms. For example, in the 32 long deep-wing trawl, relatively more netting in the wing may have been important in terms of visibility for tailor and southern herring and promoted the avoidance of some individuals. By comparison, both the shorter 32 trawls might have already been quite visible. Reducing the depth of the wing may have more readily directed some fish toward the Nordmøre-grid−which was located relatively closer to the trawl mouth. In the absence of additional data, both of the above hypotheses are speculative. Future research would benefit from a more detailed assessment of the behavior of key species in relation to the above postulated geometric trawl changes.

The potential behavior of school prawns during capture might also explain why the 32 short shallow-wing trawl caught similar (or greater) quantities as all other small-meshed trawls in Lake Wooloweyah, but was less consistent in the Clarence River. Apart from slightly different spread wing-end spreads, the only main technical difference between experiments was the otter boards, with a solid design used in all trawls in the shallow lake, compared to a 172-mm gap used with the 32 short deep-wing trawl and an 88-mm gap with all other small-meshed trawls in the river. During previous work [12], [13], we hypothesized that otter boards have a herding effect on school prawns, attributed to individuals buried in the substratum being disturbed and directed into the trawls (possibly after contacting the inner surface of the otter board). The larger gap used with the 32 short shallow-wing trawl may have allowed some school prawns to pass through the otter board after being disturbed. Any potential for such effects might be eliminated by placing mesh in the gap, although debris accumulation would increase otter-board surface area.

Irrespective of the effects on catches, reducing the twine area via less wing depth and a steeper body taper (and subsequently less otter board area in deeper water) had clear incremental impacts on drag and therefore fuel consumption. Ultimately, compared to the conventional 41 long deep-wing trawl, the 32 short shallow-wing trawl tested in the river required 18 and 12% less fuel per hour and ha trawled respectively. Based on the data presented here for the tested trawler, and assuming six hourly deployments, the fuel savings would equate to ∼13 L per day during conventional fishing or up to ∼1700 L per fishing season—a saving of ∼$A3000.

The engineering and biological results observed here have implications for on-going research. Clearly, determining the most appropriate mesh size and ideally ensuring consistent lateral openings is an important precursor to other anterior gear modifications. We showed that it is possible to considerably reduce mesh size (e.g. by 22%), but still dramatically improve species selection (i.e., reduce total bycatch by up to 57%) through other changes; presumably because mesh openings mostly determined the selectivity of the targeted school prawns, whereas teleosts were more affected by changes in trawl geometry. Based on the mean sizes of school prawns retained in both experiments, the 32-mm mesh is too small. However, it should be possible to increase mesh size slightly to somewhere less than 41 mm, while still using a steep side taper to minimize teleost bycatch, and with the least amount of twine area to reduce otter-board area and drag. These results support a similar concept of attempting to optimize mesh size as a precursor to anterior penaeid-trawl changes designed to improve environmental performances in other fisheries.
